# Practical Treatise on Military Surgery

**Published:** 1861-06

**Authors:** 


Bailliere Brothers announce the forthcoming publication of a Practical Treatise on Military Surgery, by Frank Hastings Hamilton, M. D., the eminent Surgeon and Author.

The work will embrace the Examination of Recruits, Hygiene, and Accommodation of Troops in Tents, Barracks, Huts, etc.; Hospitals; Preparations for the Field; Flying Ambulances, Litters, etc.; Gunshot Wounds, Amputations, Hospital Gangrene, Scurvy, etc.; United States Army Medical Regulations, with many other matters pertaining to Military Surgery.

